# Amphotericin B- and Levofloxacin-Loaded Chitosan Films for Potential Use in Antimicrobial Wound Dressings: Analytical Method Development and Its Application

**DOI:** 10.3390/pharmaceutics14112497

**Published:** 2022-11-17

**Authors:** Ke Peng, Mingshan Li, Achmad Himawan, Juan Domínguez-Robles, Lalitkumar K. Vora, Ross Duncan, Xianbing Dai, Chunyang Zhang, Li Zhao, Luchi Li, Eneko Larrañeta, Ryan F. Donnelly

**Affiliations:** 1 School of Pharmacy, Queen’s University Belfast, Medical Biology Centre, 97 Lisburn Road, Belfast BT9 7BL, UK; 2 Department of Pharmaceutical Science and Technology, Faculty of Pharmacy, Hasanuddin University, Makassar 90245, Indonesia

**Keywords:** amphotericin B, levofloxacin, chitosan film, wound dressing, antimicrobial

## Abstract

Levofloxacin (LVX) and amphotericin B (AMB) have been widely used to treat bacterial and fungal infections in the clinic. Herein, we report, for the first time, chitosan films loaded with AMB and LVX as wound dressings to combat antimicrobial infections. Additionally, we developed and validated a high-performance liquid chromatography (HPLC) method coupled with a UV detector to simultaneously quantify both AMB and LVX. The method is easy, precise, accurate and linear for both drugs at a concentration range of 0.7–5 µg/mL. The validated method was used to analyse the drug release, ex vivo deposition and permeation from the chitosan films. LVX was released completely from the chitosan film after a week, while approximately 60% of the AMB was released. Ex vivo deposition study revealed that, after 24-hour application, 20.96 ± 13.54 µg of LVX and approximately 0.35 ± 0.04 µg of AMB was deposited in porcine skin. Approximately 0.58 ± 0.16 µg of LVX permeated through the skin. AMB was undetectable in the receptor compartment due to its poor solubility and permeability. Furthermore, chitosan films loaded with AMB and LVX were found to be able to inhibit the growth of both *Candida albicans* and *Staphylococcus aureus*, indicating their potential for antimicrobial applications.

## 1. Introduction

Chronic, difficult-to-heal wounds are at risk of fungal infections and are also at risk of developing bacterial infections. They are generally polymicrobial in nature [1]. Various microorganisms cluster and coexist in their niche, where bacteria (*Staphylococcus spp.*) and fungi (*Candida spp*.) generally predominate the polymicrobial population [2]. Therefore, antibiotics and fungicides must be used in combination to combat those types of polymicrobial wounds [3,4]. Levofloxacin (LVX), a fluoroquinolone antibiotic, is active against a broad range of Gram-positive, Gram-negative and atypical bacteria. It has been widely used in the treatment of various infectious diseases. Although LVX is relatively safe and tolerable, gastrointestinal (GI) disturbances and stimulation of the central nervous system (CNS) have been observed [5]. Topical application of LVX could avoid GI and CNS side effects andLVX has been widely reported in the application of wound dressings [6,7,8]. Amphotericin B (AMB), a polyene fungicide, has been in clinical use for decades for treating human fungal infections, especially opportunistic systemic fungal infections [9]. It possesses broad-spectrum antifungal activities and rare antifungal resistance [10]. Systemic use of AMB has the potential for significant toxicity, especially nephrotoxicity and electrolyte abnormalities [11]. AMB has been suggested to be used topically to treat wound infections [12,13,14].

In wound management, topical application is the first choice, but several antibiotics, including AMB, suffer from low wound drug concentrations, which are needed to eradicate infections. A preliminary study reported poor wound penetration of AMB after systemic liposomal AMB administration, risking subinhibitory concentrations of the fungicide at the site of infection [13]. Another case report attempted to administer topical AMB for postoperative mucomycosis with positive outcomes observed from the wound healing process [13,15]. In contrast with AMB, LVX was reported to have good wound penetration, but considering its systemic side effects, the topical route may be beneficial to avoid adverse reactions [16,17].

Wound dressings can be enriched with antimicrobial drugs to better address the risk of developing infections during wound care [18,19,20]. Two antimicrobial agents can be combined in one preparation to resist the development of resistant pathogens [21,22]. To extend its antimicrobial properties and to better tackle polymicrobial infection, an antibacterial and antifungal drug can be combined into one preparation [3]. In this work, we use LVX and AMB where a synergistic interaction between LVX and AMB has been reported [4].

A novel dual drug-loaded wound dressing using a chitosan film to deliver AMB and LVX was designed in the study with the idea of extending the antimicrobial activities of the chitosan films. Chitosan, a natural cationic polysaccharide obtained by alkaline deacetylation of chitin, is nontoxic, biocompatible, biodegradable, mucoadhesive and antimicrobial [23]. Chitosan exerts its antibacterial activities through the binding of its cationic amino groups to anionic groups of these microorganisms [24]. These properties make chitosan an ideal vehicle for the medical field. Chitosan possesses outstanding film-forming capabilities, which are greatly appreciated in wound dressing systems, and its films are sometimes stated as ‘‘bioactive dressings’’ [23,25]. Moreover, the combined use of AMB and LVX was found to exert synergistic interactions against fungal cells, which may potentially treat concurrent bacterial and fungal infections [4]. Therefore, both bacterial and fungal infections may be treated by using only one pharmaceutical dosage form.

Method development followed by validation is an important step in the development of novel pharmaceutical preparations, especially in combination drug products. Several analytical methods have been reported to analyse AMB and LVX individually but to the best of our knowledge, no analytical method has been reported for the simultaneous quantification of AMB and LVX. Herein, we have developed and validated an HPLC-UV analytical method to fill this gap. The developed and validated method was applied to evaluate the release profile of the film, and antimicrobial performance was studied to demonstrate antimicrobial efficacy.

## 2. Materials and Methods

### 2.1. Materials

Amphotericin B (AMB) was purchased from Cayman Chemical Company (purity specification ≥ 95%), Ann Arbor, MI, USA. Levofloxacin hydrochloride (LVX) was obtained from Zhejiang Jingxin Pharmaceuticals (Xinchang, China). Plasdone™ K-29/32 (poly(vinylpyrrolidone), PVP) (MW 58 kDa) was donated by Ashland (Kidderminster, UK). Chitosan (low molecular weight), ethylenediaminetetraacetic acid disodium salt dehydrate (EDTA-Na2), dodecyl sodium sulphate (SLS) and HPLC-grade methanol were also supplied by Sigma-Aldrich (Poole, Dorset, UK). Dimethyl sulfoxide (DMSO) was provided by VWR International Limited, Leicestershire, UK. Phosphate buffered saline (PBS) pH 7.4 tablets were acquired from Oxoid Limited, Hampshire, UK. All other chemical reagents were of analytical grade and purchased from Sigma-Aldrich (Dorset, UK) or Fisher Scientific (Leicestershire, UK).

### 2.2. Film Preparation and Characterization

The films were prepared using the solvent casting method. Chitosan stock solution was prepared by dissolving 3% *w*/*v* chitosan in 1 M acetic acid and stirring overnight at 25 °C. This chitosan stock solution (5 g) was mixed with 0.10 g PVP and 0.90 g water to form the film solution. For the films containing AMB and LVX, 25 mg of AMB dissolved in 0.3 mL DMSO and 25 mg of LVX were added to the film solution and mixed using a SpeedMixer™ (DAC 150.1 FVZ-K, FlackTek, Hamm, Germany) at 3500 rpm for 3 min to form a homogenous solution. An aliquot of 250 µL of the resulting solution was added into a circular silicone mould with a diameter of 2 mm and placed in a fume hood to dry overnight. For the blank film, 0.3 mL DMSO was added to the film solution. For films containing only AMB, 25 mg of AMB dissolved in 0.3 mL DMSO was added into the film solution without the addition of 25 mg of LVX. For films containing only LVX, 25 mg of LVX with 0.3 mL DMSO was added to the film solution. The structures of the obtained films were studied by scanning electron microscopy (SEM) (Hitachi TM3030; Tokyo, Japan), a digital microscope (Leica EZ4 D, Wetzlar, Germany) and an optical coherence tomography (OCT) microscope (EX1301, Michelson Diagnostics Ltd., Kent, UK).

### 2.3. Instrumentation and Chromatographic Conditions

Simultaneous analysis of AMB and LVX was performed with an Agilent Technologies 1260 Infinity HPLC consisting of an Agilent degasser, quaternary pump, auto standard injector and detector (Agilent Technologies UK Ltd., Stockport, UK). Separation was achieved by using a C18 Phenomenex InertClone^TM^ analytical column ODS (3) (250 mm × 4.60 mm internal diameter, 5 µm packing; Phenomenex InertClone^TM^, US). The mobile phase consisted of a mixture of 2.5 mM EDTA-Na_2_ (mobile phase A) and methanol (mobile phase B). The injection volume was 20 μL, and elution was performed at a constant flow rate of 1 mL/min for 16 min. The column was thermostated at 30 °C. The detection wavelengths were set at 295 nm (for the analysis of LVX) and 406 nm (for the analysis of AMB). The obtained chromatographs were analysed using Agilent ChemStation^®^ software B.02.01.

The stock solutions were prepared by dissolving 10 mg of AMB and 10 mg of LVX together with 10 mL of DMSO and methanol (1:1, *v*/*v*), and the standard samples were prepared by diluting the stock solution using methanol. The instrumentation and chromatographic conditions for AMB and LVX are presented in Table 1. These HPLC methods were validated according to the International Council for Harmonisation (ICH) guidelines for Validation of Analytical Procedures Q2 Analytical Validation Revision one (R1) 2005 [26,27].

### 2.4. Analytical Method Validation

#### 2.4.1. Specificity

The specificity of the method was guaranteed by observing potential interferences caused by blank release medium under current chromatographic conditions without the presence of both drugs to evaluate if the matrices have interferences from other components of similar behaviour.

#### 2.4.2. Linearity and Calibration Curve

The linearity of the proposed method was assessed through calibration curves, constructed with eight standard solutions ranging from 0.7 to 5.0 µg/mL to determine the coefficient of correlation, slope and intercept values. The calibration curves were obtained by plotting the peak areas against the corresponding drug concentrations with least squares linear regression analysis using the Analysis ToolPak of Microsoft Excel^®^ (Microsoft Corp., Redmond, WA, USA).

#### 2.4.3. Detection and Quantitation Limits (LODs, and LOQs)

The theoretical LOD and LOQ for the developed methods were calculated using the standard deviation of the response (σ) and slope (*S*) of the calibration curve according to the following equations:

$$LOD=\frac{3.3\times \sigma }{S}$$

$$LOQ=\frac{10\times \sigma }{S}$$

#### 2.4.4. Precision and Accuracy 

The precision of the methods was verified using three standard concentrations of each drug (1, 2, 4 µg/mL as low, medium, high concentrations). Accuracy is defined as the closeness of agreement between the measurements and an accepted reference value, expressed as the relative error (RE). The precision is expressed as the closeness of agreement (degree of scatter) between a series of measurements, represented as the relative standard deviation (RSD) [28]. Both RSD and RE were calculated using the following equations. Accuracy and precision were evaluated intraday and interday, and the method was deemed to be accurate and precise if the RSD and RE from all samples fell below 15% [29,30].

$$RSD\;\left(\%\right)=\frac{Standard\;deviation}{Mean}\times 100\%$$

$$RE\;\left(\%\right)=\frac{Back\;calculated\;value-true\;value}{True\;value}\times 100\%$$

### 2.5. Application of the Analytical Method to the In Vitro Release Study

The release profiles of both AMB and LVX were investigated as a direct application of the analytical method. The chitosan films were placed in 10 mL of PBS (pH 7.4) with 1% *w*/*v* SLS at 37 °C and 40 rpm. SLS was added to increase the solubility of AMB in PBS (pH 7.40) to maintain a sink condition. An aliquot of 1 mL of sample was taken at 1 d, 2 d, 3 d, 4 d, 5 d, 6 d, and 7 d and replaced with a fresh release medium. The release samples were analysed using the validated simultaneous analytical method for AMB and LVX.

### 2.6. Application of the Analytical Method to Skin Deposition and Permeation Studies in Franz Cells

Skin deposition and permeation experiments were carried out using a glass vertical Franz diffusion cell setup. A piece of excised neonatal full-thickness (500 µm) porcine skin obtained from stillborn piglets was sandwiched between the donor and the receptor compartments with SC facing the donor. The receptor compartment was filled with 12 mL of PBS (pH 7.4) containing 1% *w*/*v* SLS and allowed to equilibrate for 1 h at 37 °C. To start the test, 10 µL of water was added to the skin surface, and the chitosan films containing AMB+LVX were put into the donor compartment with close contact with the skin. The receptor compartment was maintained at 37 °C and constantly stirred using a magnetic bar at 600 rpm. At 24 h, samples (1 mL) were withdrawn from the receptor compartment, filtered using a 0.45 µm syringe filter and analysed using HPLC. The AMB and LVX deposited in skin were estimated after the 24 h permeation studies. The skin was carefully removed from the Franz cell, wiped with a paper towel and cut into small pieces using surgical scissors. The skin tissue was further homogenized with 1 mL DMSO using a Tissue Lyser LT (Qiagen, Ltd., Manchester, UK) at 50 Hz for 15 min. The skin homogenate samples were centrifuged at 16,160× *g* for 20 min to collect the supernatant for further dilution and HPLC analysis.

### 2.7. Microbiological Assay

A disk diffusion test was performed to evaluate the antibacterial and antifungal properties of the films against *Staphylococcus aureus* NCTC 10788 and *Candida albicans* NCYC 610 from the National Collection of Type Cultures, Central Public Health Laboratory, Colindale Avene, London, respectively. *C. albicans* was grown and maintained on Sabouraud dextrose (SDE) agar (Oxoid, Hampshire, UK) and incubated at 37 °C for 48 h. *C. albicans* was incubated overnight at 37 °C in SDE broth at 100 rpm in an orbital shaker. After growth, *C. albicans* culture was diluted using sterile PBS (pH 7.4) to an OD_550_ value of 0.10 (approx. 6.0 × 10^5^ cfu/mL). Then, 1 mL of the diluted culture was added to 5 mL of soft SDE agar, which had been previously heated to 100 °C before being allowed to cool down to below 55 °C. This composition was then mixed using a vortex and poured onto the surface of an SDE agar plate. Similarly, *S. aureus* was inoculated overnight at 37 °C in lysogeny broth (LB) at 100 rpm in an orbital shaker. The culture was then diluted using PBS to an OD_550_ value of 0.10 (approx. 1.0 × 10^8^ cfu/mL). Subsequently, 1 mL of the diluted culture was mixed with 5 mL of soft Lysogeny agar (LA) and poured on the surface of an LA plate. The chitosan films containing both drugs (AMB+LVX), containing only one drug (AMB or LVX) and blank films were placed in the centre of the inoculated plates. The agar plates containing different film samples were then incubated at 37 °C for 24 h. The whole process was conducted under aseptic conditions, and untreated inoculated plates were used as a control (n = 4).

### 2.8. Statistical Analysis

The data are shown as the means ± standard deviation (SD) unless otherwise noted. The calculation of means, SD, %RSD, %RE, LOD, LOQ and least-squares linear regression analysis were all performed using Microsoft^®^ Excel 2019 (Microsoft Corporation, Redmond, WA, USA). Unless otherwise noted, *p <* 0.05 was used to indicate statistical significance in all cases.

## 3. Results

### 3.1. Preparation and Characterization of Topical Films

**Figure 1:**
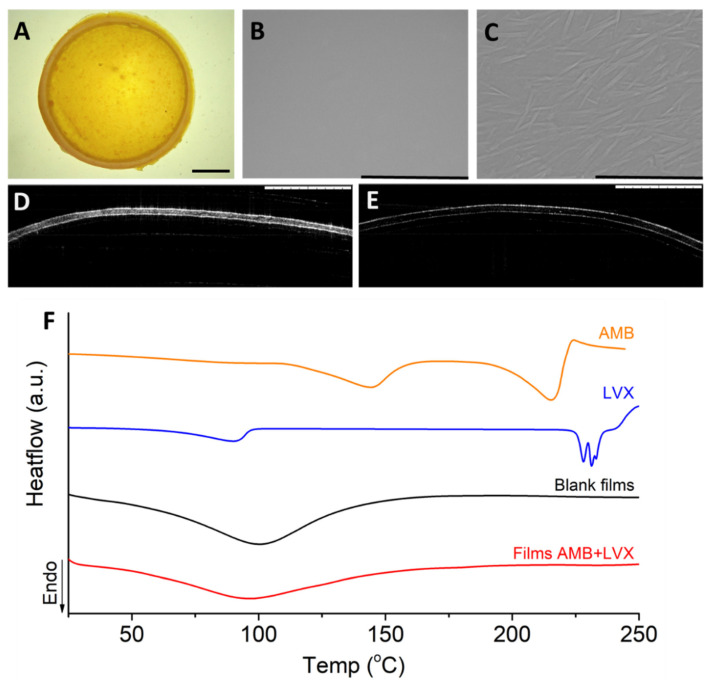
The structure of chitosan films containing AMB and LVX. (**A**) Digital microscopy. Scale bar: 2 mm. (**B**) SEM image. Scale bar, 1 mm. (**C**) SEM image. Scale bar, 100 µm. (**D**) OCT image of chitosan films containing AMB+LVX. Scale bar, 1 mm. (**E**) OCT image of blank chitosan films without AMB and LVX. Scale bar, 1 mm. (**F**) DSC thermograms of chitosan films containing AMB+LVX, blank chitosan films, AMB and LVX.

Chitosan films can be prepared using the solvent casting method, electrostatic spraying method and dry wet phase separation method, due to the film-forming capabilities of chitosan [31]. Overall, the solvent casting method is the most commonly used and most convenient method [23]. The morphology of the chitosan films we prepared using the solvent casting method was investigated using SEM [32]. As shown in Figure 1B, the films presented a smooth, flat surface without any phase separation, indicating homogenous blending between chitosan, PVP and the drugs. This finding correlates with the report in the literature [33], indicating the film forming capability of chitosan and PVP [34]. At 1000× magnification (Figure 1C), the surface was found to have crystals due to the semicrystalline nature of the polymers and the crystalline nature of the drugs [34]. To further understand the films, OCT was used to visualize the cross-section of the films. OCT is a well-established imaging tool to noninvasively map the variation in reflected light as a function of depth to indicate the cross-section information of samples [35]. As shown in Figure 1D, chitosan films containing AMB and LVX had a homogeneous thickness. The obstruction density of the chitosan films containing AMB and LVX was much higher than that of the blank chitosan films (Figure 1E), indicating a homogenous distribution of drugs inside the films. Additionally, the films were analysed using DSC. AMB is characterized by double broad endothermic peaks at approx. 144 °C and 215 °C during DSC analysis. The endothermic peak at 144 °C could be the melting point of AMB [36]. Additionally, the endothermic peak at 215 °C can be attributed to drug decomposition [37]. This is consistent with previous reports, as AMB has been reported to be a crystalline drug. Moreover, LVX shows an endothermic peak at approx. 90 °C due to the loss of free water and the drug characteristic melting point at approx. 230 °C, which demonstrates good agreement with previously reported values [7,38,39,40,41]. It is important to note that, when both drugs were incorporated into the film, these melting points were not observed. Therefore, it can be concluded that both drugs were dispersed homogenously in the film. Chitosan and PVP are stable polymers with high decomposition temperatures. The endothermic peaks observed in the blank and drug-containing films probably contributed to the residual solvent loss, including acetic acid and water, after preparation [42]. This is because chitosan is known to have a strong affinity with water [43] and PVP is hygroscopic in nature [44]. The thermogram also indicates that the films might be amorphous/semi-crystalline in nature, as PVP is known as an amorphous material [45]. PVP is a well-known pharmaceutical ingredient for its nontoxic, bioinert, and hydrophilic properties and has been widely used in wound dressings. The addition of PVP to chitosan has been reported to have little effect on the physical and chemical properties of chitosan, but can improve the mechanical properties of chitosan due to its ability to interact with PVP *via* hydrogen bond formation between the carbonyl group of PVP and the amino or hydroxyl groups of chitosan [46]. Moreover, the addition of PVP to chitosan films lowers the preparation cost, as PVP is a synthetic polymer much more affordable than the naturally occurring polymer chitosan, which requires extraction from crustacean shells [25].

### 3.2. Analytical Method Development

A C18 column was chosen in this analytical method due to its adaptability and versatility for a broad range of compounds. The HPLC analysis of AMB reported in the literature generally uses the chelating agent EDTA in the mobile phase, as EDTA could directly compete against AMB for chelation with metal ions possibly from the column packing materials, therefore improving the chromatographic peak shape [47,48]. At the beginning of the method development, an isocratic elution was applied to separate AMB and LVX. However, a high ratio (over 60%) of the aqueous phase was required to retain LVX, in which case the retention time of the AMB was over 30 min. A gradient method was, therefore, developed in this study to achieve a shorter runtime and desired sensitivity for simultaneously analysing AMB and LVX due to the huge difference between these two molecules in polarity. The wavelengths were set at both 295 nm and 406 nm in the analysis based on the maximum absorption wavelengths of individual compounds (406 nm for AMB and 295 nm for LVX) [49,50,51].

### 3.3. Method Validation

ICH recommendations were consulted to perform method validation.

#### 3.3.1. Specificity

**Figure 2:**
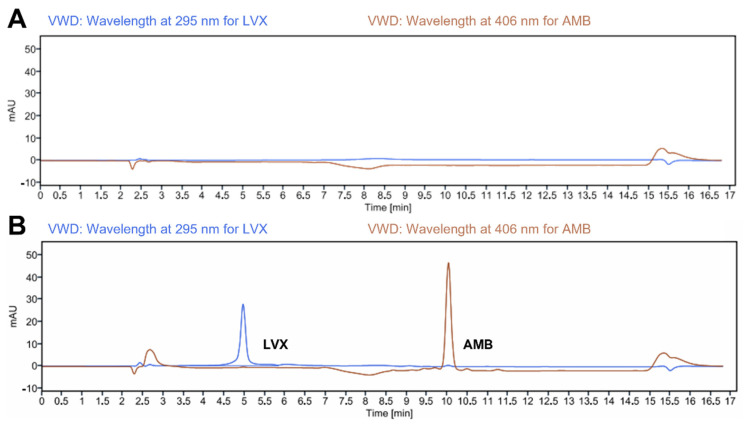
Representative chromatograms of (**A**) blank release medium (PBS pH 7.4 containing 1% *w*/*v* SLS); and (**B**) LVX and AMB (4.0 μg/mL for both drugs) in release media.

The retention time for AMB and LVX under the present experimental conditions was 10.028 (at 406 nm) and 4.965 min (at 295 nm), respectively (Figure 2B). The resolution of the two peaks is 3.74, greater than 1.5, indicating that the sample components are well-separated to an extent where the area or height of each peak can be accurately measured [52]. Representative chromatograms of the drug-free release medium (as presented in Figure 2A) showed no peaks at these retention times, demonstrating the specificity of the current analytical method.

#### 3.3.2. Linearity and Calibration Curve

The linearity of the proposed method was determined by analysing different concentration levels of both AMB and LVX and determining their integrated peak area. The peak areas were correlated to the corresponding concentrations to plot the calibration curve. Linearity and the parameters of the regression equation are listed in Table 2. The results demonstrate that the calibration curves for both drugs exhibited a linear response with a coefficient of determination (R^2^) ≥ 0.99 over the concentration range analysed. Additionally, Table 2 shows the LOD and LOQ for both drugs. These values obtained for LVX are lower than previously reported HPLC methods using UV-visible detection for the quantification of the drug in dosage forms [53]. The LOD and LOQ could be reduced by using more sensitive detectors, such as fluorescence detectors or higher-end equipment, such as UPLC [54,55,56]. However, these methods were developed in many cases for the quantification of LVX in biological fluids requiring low drug concentrations. On the other hand, AMB methods described in the literature are focused on the quantification of the drug on biological matrices and therefore are not suitable for formulation development, as they require complex extraction procedures or the use of high-end equipment such as mass spectrometry [57,58].

#### 3.3.3. Accuracy and Precision

The accuracy and precision results for the QC samples are reported in Table 3. In terms of accuracy, the values of %RE were found to be within −6.02 to 5.63%, falling within the required limits of 15%, demonstrating that the current analytical method is accurate [29]. In regard to precision, the values of %RSD were found to be within 1.62 to 5.06%, falling within the required limits of 15%, showing that the current analytical method is precise [30,59].

### 3.4. Release of Topical Films

**Figure 3:**
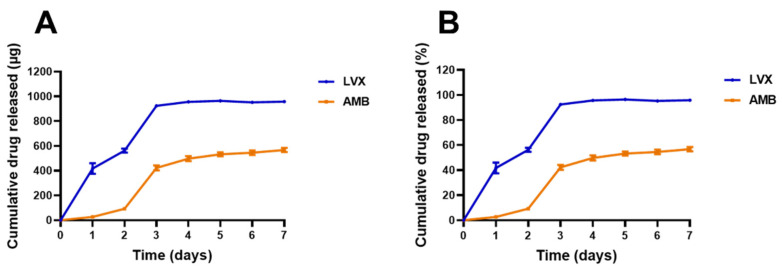
Release profiles of AMB and LVX in PBS (pH 7.4) containing 1% *w*/*v* SLS over seven days. Cumulative drug released in µg (**A**) and percentage (**B**). (Means ± SD, n = 5).

The release of AMB and LVX from the chitosan films was evaluated and quantified using the analytical method validated above, and the release profiles are shown in Figure 3. The amount of AMB and LVX was kept the same at 1.00 mg per disc to study the release profiles of each drug at the same level. The release of AMB and LVX from chitosan films followed a similar pattern: they were released quickly in the first three days and reached a plateau by seven days. However, compared to AMB, LVX was released more quickly and more completely from the chitosan films. It released 924.8 µg (92%) by the third day and 958.5 µg (96%) by the seventh day. In the case of AMB, its release reached 422.8 µg (42%) by the third day and increased gradually afterward to 567.5 µg (57%) on the seventh day. These similar patterns could be attributed to the same shape and fabrication materials of the films. The different release phenomena could be explained by the different properties of the drugs. LVX is a water-soluble compound that can easily dissolve and enter the release medium by free diffusion, thereby presenting a more complete release after 3 days [60]. However, AMB is practically insoluble in water at neutral pH values. In the PBS environment (pH 7.4), the release of AMB from chitosan films was found to be slow. Similar release profiles have been demonstrated in the literature, with AMB being released slowly from chitosan particles [61].

### 3.5. Skin Deposition and Permeation Studies in Franz Cells

**Figure 4:**
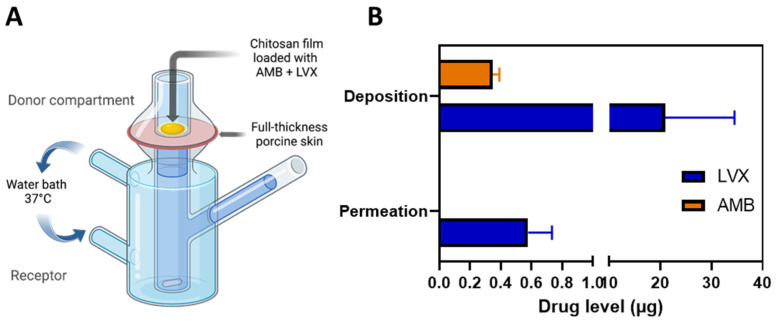
Skin permeation and deposition results. (**A**) Schematic representation of the modified Franz cell setup for the ex vivo skin permeation and deposition study. The chitosan films containing AMB+LVX were placed on top of the full-thickness porcine skin and 10 µL of water was added between the film and skin to facilitate adhesion and mimic the moist environment of the wound tissue. The skin tissue was homogenized to extract the drug deposited and the samples from the receptor compartment were analysed for permeation data. (**B**) Ex vivo permeation and deposition results of AMB and LVX from the chitosan films. (Means + SD, n = 3).

Ex vivo skin deposition and permeation experiments were performed on modified Franz cell setup (Figure 4A) using excised neonatal full-thickness porcine skin because of its remarkable similarity in general structure and physical characteristics to human skin [62,63]. Water (10 µL) was added to facilitate adhesion between the film and the skin as well as to mimic the moist environment of the wound tissue. As shown in Figure 4B, after 24-hour of application, 20.96 ± 13.54 µg of LVX was deposited in the skin and approximately 0.58 ± 0.16 µg of LVX could permeate from the skin to the receptor compartment. Approximately 0.35 ± 0.04 µg of AMB was deposited in the skin and little amount of AMB was able to reach the receptor compartment. These data showed a very limited permeation profile of AMB and relatively better profiles of LVX. This has been well documented in the literature [13,16,17]. This could be attributed to the different characteristics of the drugs [64]. LVX is classified as a BCS class I drug, which possesses high solubility and high permeability [65]; while AMB is characterized as a BCS class IV drug, which is notorious for its low solubility and low permeability [66]. The MIC of LVX against *S. aureus* is reported to be in the range of 0.06–0.5 mg/L [67,68,69] The skin deposition and permeation of LVX from the film was high enough to inhibit the growth of *S. aureus* deep in the skin layer. The MIC of AMB against *Candida* is reported to be mostly around 0.25–1 mg/L [70]. The deposition of AMB reached 0.35 ± 0.04 µg, indicating the applicability of the chitosan films to treat superficial fungal infections. These data indicated that the chitosan films we developed in this study could serve for localized topical use as the systemic exposure (indicated by the permeation data) is likely to be minimal, indicating good safety profiles, especially for toxic drugs such as AMB [71]. Even though there are very limited resources to define concentration toxicity threshold for levofloxacin [72,73], the safety profile of levofloxacin has been widely reported in the literature and a high daily dose of 1000 mg is recommended for patients [74]. The deposition and permeation of LVX from the film (approximately 21.54 µg in total) should be tolerable [75]. 

### 3.6. Antimicrobial Efficacy

Chitosan films, with or without drugs, were evaluated for their antimicrobial effects against both microorganism strains (*C. albicans* NCYC 610 and *S. aureus* NCTC 10788). The disk diffusion test here was used to represent the overall antimicrobial ability of the films and a complementary examination to confirm the release of both drugs. *C. albicans* was used as a representative fungus because it is commonly found on the skin surface and mucous membranes and is the most common cause of invasive fungal infections coinciding with mortality rates as high as 40% [76,77]. Moreover, *S. aureus* is a Gram-positive bacterium that can cause a wide variety of clinical diseases. *S. aureus* is a good example of a pathogen that is involved in both community-acquired and hospital-acquired infections [78,79]. 

**Figure 5:**
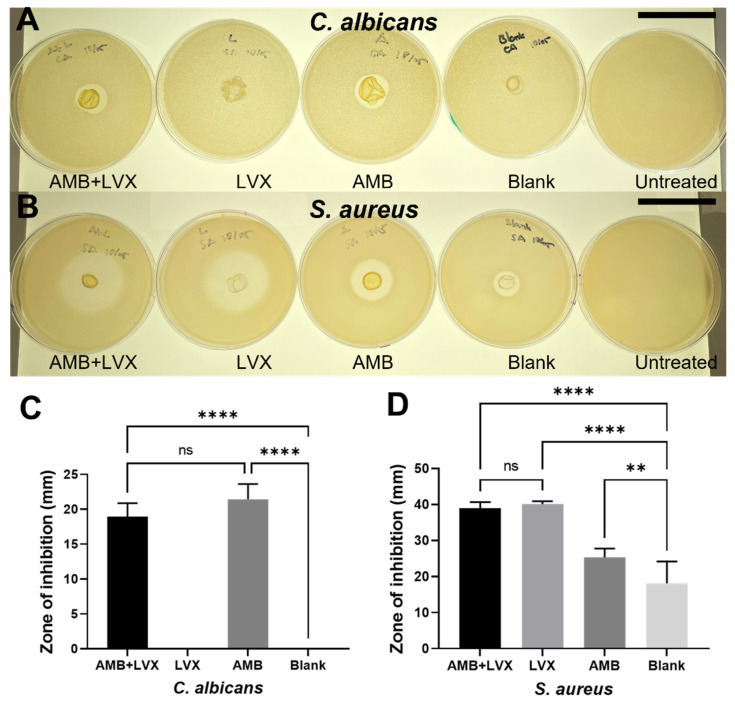
Antimicrobial performances of chitosan films containing AMB and LVX. (**A**) Representative disk diffusion test pictures against *C. albicans* after exposure to chitosan films containing AMB and LVX, chitosan films containing LVX, chitosan films containing AMB, blank chitosan films and untreated control. Scale bar, 5 cm. (**B**) Representative disk diffusion test results against *S. aureus* after exposure to chitosan films containing AMB and LVX, chitosan films containing LVX, chitosan films containing AMB, blank chitosan films and untreated control. Scale bar, 5 cm. (**C**) Disk diffusion test results of chitosan films with or without AMB or LVX against *C. albicans* (means + SD, n = 4). (**D**) Disk diffusion test results of chitosan films with or without AMB or LVX against *S. aureus* (means + SD, n = 4; ns: not significant, ** *p <* 0.01, **** *p <* 0.0001).

Chitosan films containing both AMB + LVX were found to be effective against both *C. albicans* (inhibition zone of 18.93 ± 1.95 mm) and *S. aureus* (inhibition zone of 38.89 ± 1.76 mm) (Figure 5). Furthermore, chitosan films containing only LVX showed no inhibition against *C. albicans*. However, these samples presented considerable inhibition against *S. aureus* (inhibition zone of 40.10 ± 0.79 mm). Chitosan films containing only AMB drug not only exhibited a clear inhibition against *C. albicans* (inhibition zone of 21.46 ± 2.2 mm) but also against *S. aureus* (inhibition zone of 25.30 ± 2.40 mm). Additionally, although blank chitosan films (no loaded drugs) showed no inhibition against *C. albicans*, these films showed a clear inhibition against *S. aureus* (inhibition zone of 18.15 ± 5.98 mm).

With regard to antifungal activities, blank chitosan films and chitosan films containing only LVX failed to inhibit the growth of *C. albicans*. However, AMB-loaded chitosan films, including both chitosan films with AMB+LVX and chitosan films loaded only with AMB, offered substantial antifungal activities, although no significant differences in terms of inhibition zone diameter between these two samples were found (*p >* 0.05). These results demonstrated that LVX, chitosan or PVP had no meaningful effect on inhibiting the growth of *C. albicans*, and thus AMB was the main cause of the fungicidal activities from AMB-loaded chitosan films. Chitosan has well-known antibacterial activities against bacteria. However, its effects on fungal cells depend on several factors, including molecular weight, the length of the polymeric chains and variations in pH and concentration [80,81,82]. It has been reported that blank chitosan films did not show any inhibitory effects on the *Candida* species, including *C. albicans*, *Candida tropicalis* and *Candida parapsilosis* tested in the study conducted by Oliveira et al. [81]. 

In terms of their antibacterial activities, chitosan films themselves presented a modest reduction in *S. aureus* growth. This antibacterial behaviour could also be attributed to the possible existence of acetic acid in the formulation. The antibacterial ability of acetic acid has been widely applied in wound management for a long time as a disinfected and antiseptic agent [83]. Although corrosive at concentrations between 10%–30%, acetic acid is considered harmless below concentrations of 5% [84]. The residue acetic acid should be within the safety range after evaporation overnight as demonstrated by the DSC result in Figure 1F: no obvious endothermic peak is present at 118 °C at the melting point of acetic acid. Furthermore, AMB-loaded chitosan films showed an inhibition zone of 25.30 ± 2.40 mm against *S. aureus*, which presented a significant difference (*p <* 0.01) compared with the results found for blank chitosan films. Therefore, these results indicate that AMB may have notable bactericidal effects against *S. aureus*, which is consistent with the results found in the literature [85]. Moreover, both chitosan films containing only LVX or containing LVX in combination with AMB showed the greatest zone of inhibition against *S. aureus* and no significant differences were found between these two film samples (*p >* 0.05). To summarize, chitosan films containing both AMB+LVX were able to inhibit the growth of both microorganisms, *C. albicans* and *S. aureus*, tested in this study.

## 4. Conclusions

This study developed, for the first time, a chitosan film loaded with AMB and LVX for wound dressing. A simultaneous quantification method for AMB and LVX using HPLC-UV was developed, validated and found to be accurate, precious and linear based on ICH guidelines. The in vitro release profiles were examined using the validated method over seven days. Ex vivo skin deposition and permeation studies were performed to further understand the utility of the film and the applicability of the method. Antimicrobial tests demonstrated the antibacterial and antifungal effects of the chitosan films containing both drugs. This study provides some insightful and preliminary foundation for the combined formulation of these two well-established drugs.

## Figures and Tables

**Table 1:** Gradient parameters for simultaneous analysis of AMB and LVX.

Time (min)	Mobile Phase A, % (2.5 mM EDTA-Na_2_)	Mobile Phase B, % (Methanol)
0	65	35
4	35	65
5	15	85
12	15	85
12.01	65	35
16	65	35

**Table 2:** Calibration parameters and sensitivity of AMB and LVX (n = 3).

Drug	Concentration Range (µg/mL)	Slope	Y-Intercept	Correlation Coefficient (R^2^)	LOD (µg/mL)	LOQ (µg/mL)
AMB	0.7–5	123.99	−8.64	0.999	0.20	0.62
LVX	0.7–5	92.16	−16.74	0.999	0.16	0.48

**Table 3:** Intraday and interday accuracy and precision of AMB and LVX (Means ± SD; n = 3).

Drug	Spiked Concentration (µg/mL)	Calculated Concentration (µg/mL)	Precision (RSD%)	Recovery (%)	Accuracy (RE%)
AMB	intraday				
	1	1.05 ± 0.04	4.08	105.12	5.12
	2	2.03 ± 0.04	2.00	101.55	1.55
	4	3.99 ± 0.17	4.35	99.78	−0.22
AMB	interday				
	1	1.01 ± 0.02	1.77	100.93	0.93
	2	1.98 ± 0.03	1.62	98.91%	−1.09
	4	3.89 ± 0.20	5.05	97.19	−2.81
LVX	intraday				
	1	1.00 ± 0.05	5.06	100.21	0.21
	2	1.91 ± 0.04	2.00	95.31	−4.69
	4	4.23 ± 0.12	2.86	105.63	5.63
LVX	interday				
	1	1.00 ± 0.05	5.02	100.02	0.02
	2	1.88 ± 0.04	2.07	93.98	−6.02
	4	3.92 ± 0.20	5.01	98.11	−1.89

## Data Availability

Not applicable.
